# Hepatic Natural Killer Cells: Organ-Specific Sentinels of Liver Immune Homeostasis and Physiopathology

**DOI:** 10.3389/fimmu.2019.00946

**Published:** 2019-04-30

**Authors:** Joanna Mikulak, Elena Bruni, Ferdinando Oriolo, Clara Di Vito, Domenico Mavilio

**Affiliations:** ^1^Unit of Clinical and Experimental Immunology, Humanitas Clinical and Research Center, Milan, Italy; ^2^Department of Medical Biotechnologies and Translational Medicine, University of Milan, Milan, Italy

**Keywords:** tissue immunity, liver, Natural Kill cell, homeostais, homeostasis

## Abstract

The liver is considered a preferential tissue for NK cells residency. In humans, almost 50% of all intrahepatic lymphocytes are NK cells that are strongly imprinted in a liver-specific manner and show a broad spectrum of cellular heterogeneity. Hepatic NK (he-NK) cells play key roles in tuning liver immune response in both physiological and pathological conditions. Therefore, there is a pressing need to comprehensively characterize human he-NK cells to better understand the related mechanisms regulating their effector-functions within the dynamic balance between immune-tolerance and immune-surveillance. This is of particular relevance in the liver that is the only solid organ whose parenchyma is constantly challenged on daily basis by millions of foreign antigens drained from the gut. Therefore, the present review summarizes our current knowledge on he-NK cells in the light of the latest discoveries in the field of NK cell biology and clinical relevance.

## Introduction

The liver is the largest solid organ in our body receiving every day more than 2,000 liters of blood from dual blood supply. Nearly 80% of blood derive from the gastrointestinal tract via the portal vein, thus being constantly filled of large amounts of foreign antigens. The remaining 20% of blood is supplied from the hepatic artery that together with the portal vein terminates into the capillary system of the liver, sinusoids, and leaves liver parenchyma through the hepatic vein.

This large inflow of antigens makes the liver an important immunological organ in which a unique microenvironment shapes both innate and adaptive immune responses in order to maintain a correct balance between immune tolerance and immune activation (1, 2). Dysregulation of immune cells in the liver is critical in the pathogenesis of several hepatic diseases, including viral hepatitis, autoimmune disorders and tumors. Liver immune compartment consist in diverse innate populations such as Natural Killer (NK) cells, Natural Killer T (NKT) cells, gamma delta (γδ) T cells, and adaptive lymphocytes, such as αβ T cells and B cells (1, 3). On the other hand, liver parenchyma is composed by hepatocytes that represent two-thirds of the total liver cells. Other non-parenchymal cells include liver sinusoidal endothelial cells (LSECs), Kupffer cells (KCs) (i.e., liver-resident macrophages), cholangiocytes, biliary cells, and hepatic stellate cells (HSCs) (3).

**Figure 1:**
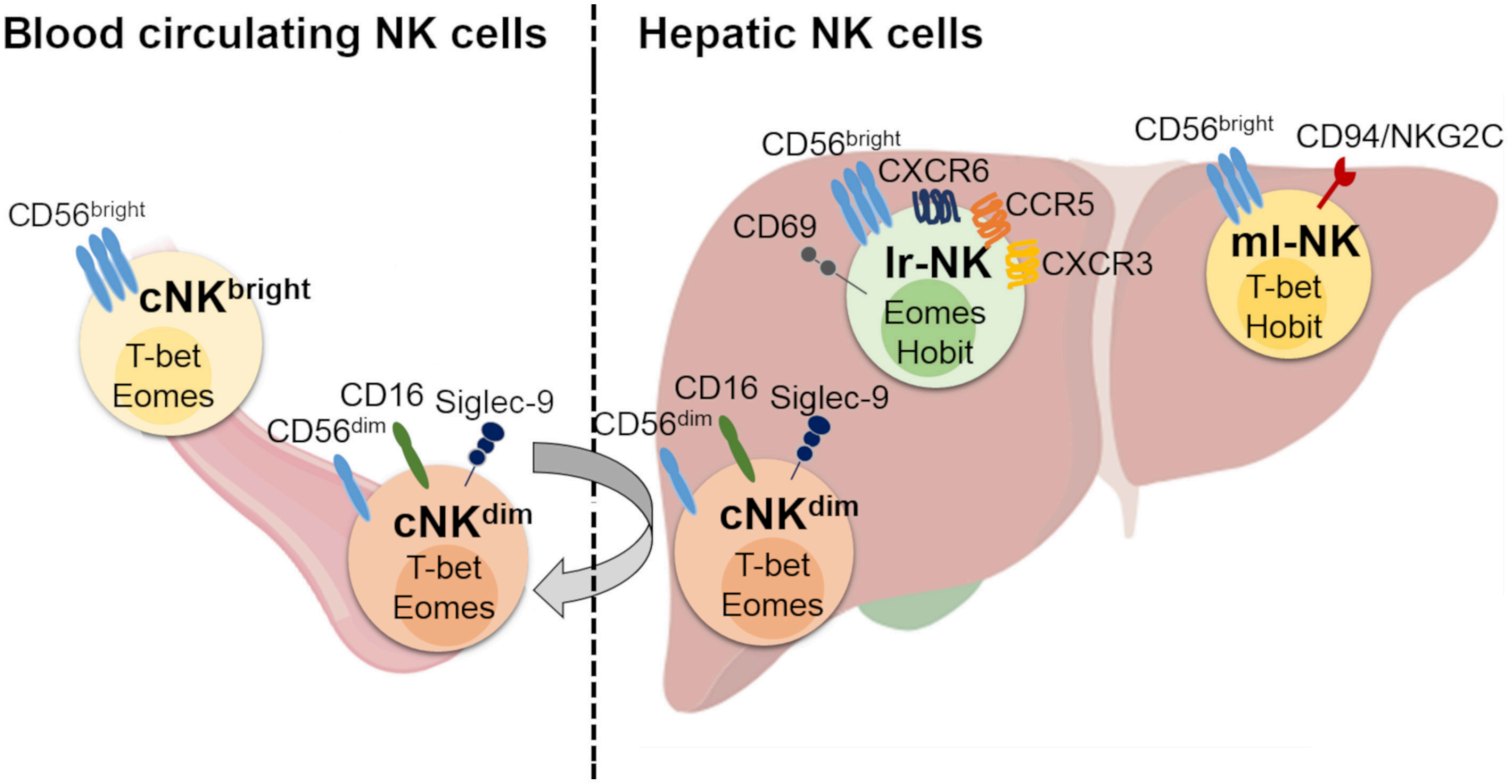
Human hepatic and conventional NK cell subsets, distribution, and phenotype. Human blood circulating conventional NK (cNK) cells contain two main subsets: CD56^dim^ (cNK^dim^) and CD56^bright^ (cNK^bright^) cells. Human hepatic NK (he-NK) cell compartment contains: liver resident NK (lr-NK) cells, memory-like NK (ml-NK) cells and transient conventional NK (cNK) cells mainly represented by recirculating cNK^dim^ cells through the liver blood system (gray arrow). Lr-NK and ml-NK cell subsets show transcriptional and phenotypic differences compared to the conventional cNK cell subsets.

Among the immune compartment, hepatic NK (he-NK) cells that contain both liver resident (lr-NK) or either transient through the adult liver conventional NK (cNK) cells, are particularly abundant and can account up to 50% of total hepatic lymphocytes (Figure 1). These innate immune effectors play key roles in order to retain a certain degree of unresponsiveness to “non-self” antigens, while are ready to attack and eliminate the true dangers to the host (2, 4, 5). Since their discovery in the early 1980s, NK cells have been valued for rapid recognition and clearance of viral-infected, tumor-transformed and stressed cell targets in the absence of antigen specificity (6). Cytotoxicity and interferon(IFN)-γ production represent the main effector-functions of mature NK cells and are controlled by a dynamic balance exerted by an array of inhibitory (iNKRs) and activating (aNKRs) receptors differently expressed on the cell surface (7). The dominant mechanism regulating the priming and activation of resting NK cells is based on the engagement of several iNKRs that bind several alleles of the major histocompatibility complex class I (MHC-I) expressed on the surface of autologous cells. This recognition spares from NK cell killing all “self” targets, thus ensuring a perfect NK cell tolerance toward our own cells. These iNKRs include inhibitory Killer Ig-like receptors (iKIRs) that recognize classical MHC-I alleles and the C-type lectin like receptor NKG2A that forms a heterodimer with the CD94 molecule (CD94/NKG2A), binding HLA-E, a non-classical MHC-I complex (8, 9). Either the decreased expression or the absence of MHC-I on target cells triggers NK cell killing, a phenomenon known as “missing-self hypothesis,” via the employment of aNKRs that, in turn, bind their putative ligands expressed on viral-infected, malignant or stressed cells. The Natural Cytotoxicity Receptors (NCRs) NKp30, NKp46 and NKp44, the C-type lectin receptors NKG2D and CD94/NKG2C heterodimer, DNAM-1, SLAM family receptors such as 2B4, and activating KIRs (aKIRs) are the main aNKRs inducing NK cell cytotoxicity (7, 10, 11).

Under homeostatic conditions, human circulating cNK cells represent about 5–15% of circulating lymphocytes and are subdivided into two main subsets defined on the basis of their differential expression of CD56 and CD16, namely CD56^bright^/CD16^neg^ (CD56^bright^) and CD56^dim^/CD16^pos^ (CD56^dim^) NK cells (12). It is largely accepted that CD56^bright^ NK cells are the precursors of the more mature CD56^dim^ NK cells, however, the developmental relationship between the different types of human NK subsets has not been finally clarified (13, 14). In this context, a recent study proposes for two CD56^bright^ and CD56^dim^ NK cell subsets distinct ontologies (14). The low amounts of intracellular cytotoxic granules (i.e., Perforin and Granzymes A-B) parallel the poor cytotoxic potential of CD56^bright^ NK cells that are also unable to perform the antibody (Ab)-dependent cellular cytotoxicity (ADCC) in line with their undetectable expression of CD16 (12). On the other hand, this latter subpopulation exerts important regulatory functions through secretion of chemokines and pro-inflammatory cytokines [i.e., IFN-γ, Tumor Necrosis Factor(TNF)-α] in response to different stimuli [i.e., interleukin (IL)-1β, IL-2, IL-12, IL-15, and/or IL-18] delivered by surrounding cells at tissue sites [i.e., macrophages, dendritic cells (DCs), and T lymphocytes] (6, 15, 16). CD56^bright^ NK cells likely give rise to terminally differentiated CD56^dim^ NK cells as they represent the largest population in blood (up to 90%), mostly express lower levels of NKp46 and CD94/NKG2A and higher amounts of iKIRs and CD94/NKG2C heterodimer (17). This population exerts both high cytotoxicity and ADCC given its high constitutive expression of CD16 in response to activation by IL-12, IL-15, and IL-18 (18). Moreover, it has been reported that CD56^dim^ NK cells can also rapidly produce IFN-γ in response to stimulation with IL-2 and/or IL-15 (19). Hence, CD56^bright^ and CD56^dim^ NK cell subsets fulfill distinct roles in immunity, with the first one serving more as immune-modulator and the second population acting mainly as cytotoxic effector. An additional NK cell subset identified on the basis of CD56 and CD16 surface expression is represented by anergic CD56^neg^/CD16^pos^ (CD56^neg^) cells that are present at very low frequency under physiologic conditions, while pathologically expanded during the course of several disorders, such as viral infections and autoimmune diseases (20, 21). More recently, an unconventional population of CD56^dim^/CD16^neg^ (unCD56^dim^) NK cells has been described. This latter subset can exert potent cytotoxicity and is extremely rare in healthy donors, while representing the main cNK cell population in the first weeks after haploidentical hematopoietic stem cell transplants (haplo-HSCT). This high frequency of unCD56^dim^ NK cells in aplastic patients affected by hematologic malignancies and receiving haplo-HSCT suggests novel pathways of NK cell ontogenesis and differentiation that are currently being investigated (22, 23).

The distribution of NK cell subsets in human tissues is very peculiar and differs from what we observe in peripheral blood. Notably, CD56^dim^ NK cells are found in high amounts in bone marrow, lung, spleen, subcutaneous adipose tissue and breast tissue. Instead, CD56^bright^ NK cells are present at high frequency in lymph nodes, gut, liver, uterus, visceral adipose tissue, adrenal gland and kidney (24, 25). Hence, other than the phenotypic and functional diversities of these latter two subsets in peripheral blood, the spectrum of human NK cell populations in tissues is much broader and likely depends on specific imprinting given by local microenvironments and by the chronic exposure to foreign antigens/inflammatory stimuli.

Here, we review our current knowledge in regard to he-NK cells with a particular focus on the breadth and generation of he-NK cell heterogeneity, under both homeostatic conditions and during the course of liver diseases.

## Heterogeneity of Liver Resident NK Cells

The liver is populated by both transient cNK cell subsets and lr-NK cells that are phenotypically and functionally distinguished (Figure 1). The first identification of tissue resident NK cells occurred in murine livers and rapidly expanded in other tissues (26). Lr-NK cells soon displayed heterogenous phenotypic profiles in different species with unique anatomical identities that reflect the impact of this peculiar tissue-niche in generating either cytotoxic or tolerogenic lymphocytes.

Murine lr-NK cells carry a CD49a^pos^/DX5^neg^ phenotype that differs from CD49a^neg^/DX5^pos^ cNK cells in mouse (27, 28). It is still unclear how the development and differentiation of lr-NK cells is regulated, but this latter subset seems to be more terminally differentiated as it lacks or has decreased expression of CD11b, Ly49, CD43, and KLRG1 (i.e., surface markers present on mature cells) compared to murine cNK cells (27). Several experimental findings indicated that lr-NK and cNK cells likely develop from separated innate lymphoid cell (ILC) lineages (29). Moreover, a common ILC progenitor subset able to differentiate in lr-NK cells but not in cNK cells has been described (30). Indeed, lr-NK and cNK cells rely on different transcriptional factors for their development, since the presence of T-bet deficiency in mice is associated with the depletion of lr-NK cells, while Eomes is critical for the maintenance of cNK cell homeostasis (Table 1) (31, 32). More recently, the new transcription factors Hobit and the aryl hydrocarbon receptor (AhR) have been reported to induce the development of different tissue-resident NK cells, including lr-NK (33, 34).

**Table 1:** Comparison of NK cell subsets in humans and mice.

**Species**	**Subset**	**Phenotype**	**Precursors**	**Transcription factors**	**References**
*Mouse*	cNK	CD49a^neg^DX5^pos^	Lin^neg^CD27^pos^CD107^neg^CD244^pos^CD122^pos^IL7Ra^neg^	Eomes	(28–33)
*Mouse*	lr-NK/ml-lr-NK	CD49a^pos^DX5^neg^	PLZF^pos^Lin^neg^IL7Ra^pos^cKit^pos^a4b7^pos^	T-bet, PLZF, Hobit, AhR	(28–35)
*Human*	cNK^dim^	CD56^dim^CD16^pos^	CD56^bright^CD94^pos^NKp80^pos^CD16^neg^CD57^neg^	T-bet, Eomes	(13, 14)
*Human*	cNK^bright^	CD56^bright^CD16^neg^	Lin^neg^CD34^neg^CD117^pos^CD94^neg^CD16^neg^	T-bet, Eomes	(13, 14)
*Human*	lr-NK^bright^ ml-NK^bright^	CCR5^pos^CXCR6^pos^CD69^pos^ CXCR6^pos^CD94/NKG2C^pos^	CD56^bright^Eomes^low^cNK^bright^ lr-CD49a^pos^CD56^bright^	Eomes^high^, Hobit T-bet, Hobit	(28, 29, 36–42)

Human lr-NK cells were first described in 1976 and were originally called “pit cells.” Only later, they were defined as highly cytotoxic NK cells resident in the hepatic sinusoids (35, 43, 44). Differently from murine and their human counterparts in peripheral blood, CD56^dim^ and CD56^bright^ NK cells are present at similar frequencies in liver and the latter subset likely corresponds to the murine CD49a^pos^/DX5^neg^ lr-NK cells, as they both share the same transcriptional factor T-bet and are negative for Eomes (Table 1) (28). However, CD49a^pos^/CD56^bright^ lymphocytes account for only 3% of all humans he-NK cells and do account for all CD56^bright^ lr-NK cells. In this regard, it is well known that several murine NK cell markers are not phylogenetically conserved in their human counterparts and this largely explains the absence of a phenotypic match between murine and human lr-NK cells. Only recently, the phenotype of human CD56^bright^ lr-NK cells has been better characterized by disclosing their constitutive expression of the chemokine receptors CXCR6 and CCR5 and of the tissue-residency marker CD69 (28, 45, 46). As a matter of fact, these 3 surface markers are absent on CD56^dim^ he-NK cells. Hence, the CD56^bright^/CCR5^pos^/CXCR6^pos^/CD69^pos^ phenotype identifies human lr-NK cells that also appear to be more heterogeneous in their development pathways compared to murine counterparts as they express high levels of Eomes transcripts rather than T-bet (36, 45, 46). Indeed, only those 3% of human CD49a^pos^/CD56^bright^ lr-NK cells resulted positive for T-bet, while the transcription factor Hobit was found positive on all CD56^bright^ lr-NK cells (28, 37).

Very little is known about the mechanism(s) regulating both recruitment and retention of NK cells in the liver. Within the hepatic microenvironment, NK cell interactions with LSECs certainly play a key role, as the masking of CD2, CD11a, CD18, and CD54 (ICAM-1) with neutralizing monoclonal Abs (mAbs) block their recruitment to the liver (38). Moreover, the constitutive high surface levels of CXCR3, CXCR6, and CCR5 on lr-NK cells are important in the retention of these hepatic lymphocytes. Indeed, the engagement of these chemokine receptors following the binding with their cognate ligands (i.e., CCL3, CCL5 and CXCL16, respectively) expressed by cholangiocytes, LSECs, hepatocytes, and KCs, is associated with liver homing (45, 47). Sinusoidal endothelial cells also express VAP-1 that, in turn, binds Siglec-9 expressed on cNK cells, thus mediating their migration to the liver (47, 48). This latter pathway seems a mechanism restricted to hepatic trafficking, since VAP-1^pos^ cNK cells do not express L-selectin (CD62L) and CCR7 receptor required for homing in secondary lymphoid tissues (27).

Another important question is whether he-NK cells stably reside in the liver or recirculate through liver sinusoids. Experimental evidence obtained from human transplanted liver revealed that Eomes^high^ lr-NK cells can persist for decades, thus further supporting the idea that these cells represent a long-lived tissue-resident subset (49). In addition, CD56^bright^/Eomes^low^ cNK cells recruited to the liver have the potential to become CD56^bright^/Eomes^high^ NK cells. This last piece of data suggests that cNK cells can also represent precursors of their liver-resident counterparts, although the associated mechanisms involved in this process have not yet been disclosed (49). Interestingly, the administration of an anti-α4β1 and -α4β7 integrins mAb (i.e., natalizumab) in patients with multiple sclerosis is associated with an remarkable increased frequency of NK cells in peripheral blood, thus indicating that they can migrate across the tissue endothelial barriers including the hepatic ones (39, 50). However, it is important to highlight that he-CD56^dim^ NK cells are transcriptionally and phenotypically similar to their circuiting counterparts and this evidence indicates that they likely recirculate through the liver blood system without being retained in the organ. This is not the case for lr-CD56^bright^ NK cells that are also transcriptionally different from their homologs in the peripheral blood (45).

The liver is also home of peculiar and newly identified lr-NK cells endowed with unique adaptive traits and showing hapten-specificity (51, 52). The phenotype of these so-called “memory like” NK (ml-NK) cells in mice is CD49a^pos^/DX5^neg^ and matches with murine lr-NK cells (51, 52). It has been also shown that CXCR6^pos^ he-NK cells can retain an unconventional immunologic memory versus viral antigens including inactivated vesicular stomatitis virus (VSV), human immunodeficiency virus (HIV) and influenza (53, 54). Most of the studies characterizing human ml-NK cells focused their investigation on cytomegalovirus (HCMV) infection, that induces the expansion of “specific” CD94/NKG2C^pos^ NK cells able to produce a higher amount of IFN-γ when these “adaptive” NK cells are re-challenged with the same virus (40, 41, 55). Interestingly, it has been reported that the small subset of CD49a^pos^/CD56^bright^ lr-NK cells is characterized by a clonal-expansion of NK cells expressing CD94/NKG2C heterodimer (28). However, the existence of a specific viral-antigen recognized by a given NKRs expressed on human ml-NK cells is still being debated and never formally demonstrated. This is indeed a very hot research topic in the field of NK cell homeostasis that requires further experimental evidence and investigations.

Lr-NK cells are characterized by different features and can kill different targets as well as secrete cytokines. They have higher intracellular amounts of lytic granules (i.e., Granzymes and Perforin) and stronger cytotoxic potentials compared to their circulating counterparts (2, 42, 56). In particular, lr-NK cells are characterized by higher constitutive expression of TRAIL and FasL compared to cNK cells, thus suggesting that the tissue resident subset employs different mechanisms to eliminate targets (i.e., apoptosis) (57). Moreover, both cNK cells and lr-NK cells are able to secrete large amount of IFN-γ, but the latter population is much more efficient in the production of TNF-α and Granulocyte-macrophage colony-stimulating factor (GM-CSF), and in case of murine lr-NK cells IL-2, all key players in inflammatory responses at tissue sites (31, 32, 57).

The need of keeping an optimal degree of immune-tolerance vs. foreign antigens while ensuring a correct immune-surveillance against potential threats (i.e., infections, tumors, aberrant inflammation, and autoimmunity) certainly explains the particularly high level of heterogeneity and complexity of he-NK cells. Indeed, these features are peculiar of the liver microenvironment that is able to induce the non-conventional “long-lived” and “memory like” innate immune effectors also within NK cell compartment.

## NK Cells in Liver Tolerance and Homeostasis

**Figure 2:**
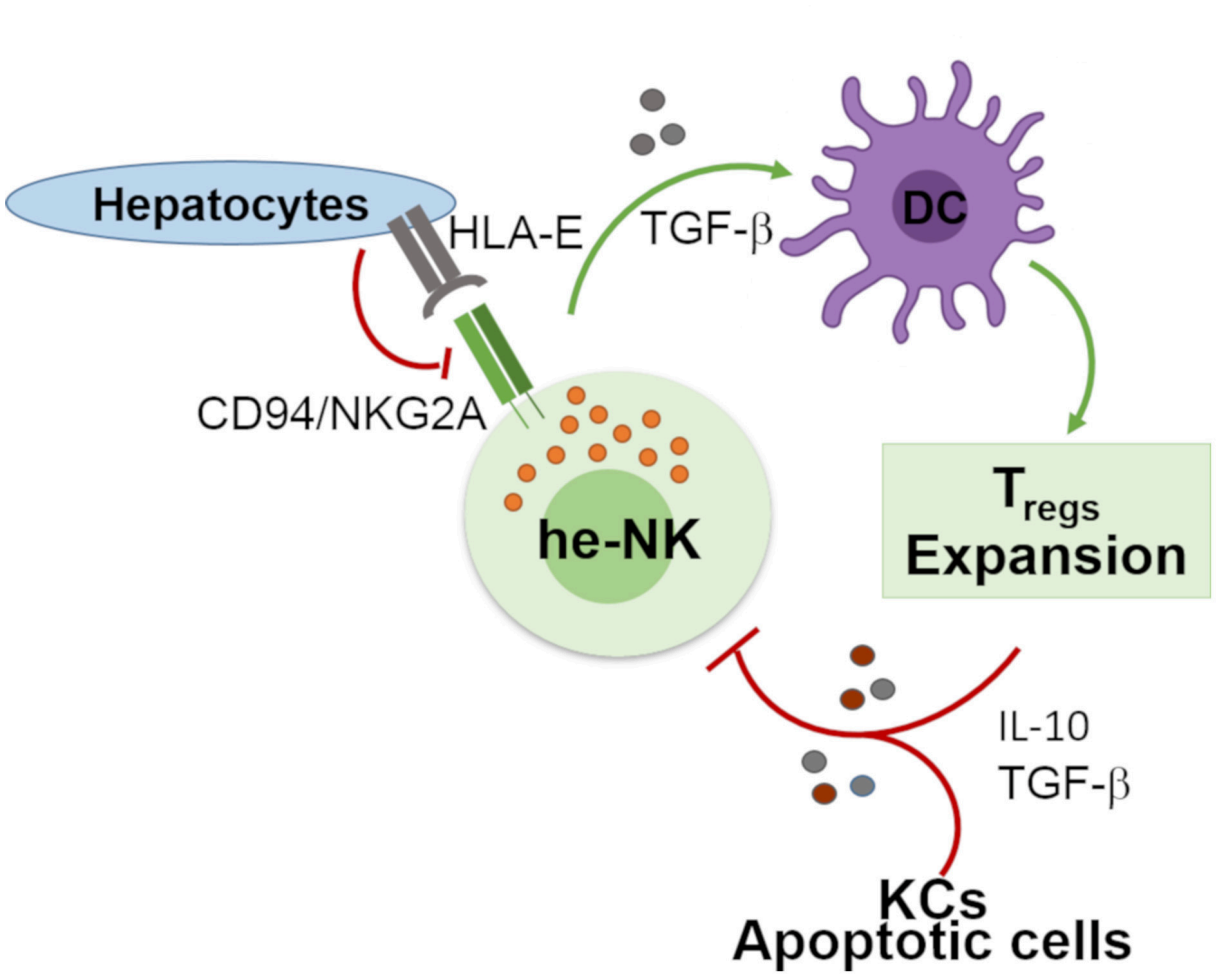
Involvement of he-NK cells in the maintenance of hepatic tolerance and homeostasis. NK cells promote hepatic tolerance by interplaying with hepatocytes via CD94/NKG2A that in a TGF-β-mediated manner modulate DCs that further prompt expansion of tolerogenic CD4^pos^CD25^pos^ Treg cells. On the other hand, Treg cells along with hepatic KCs and apoptotic cells contribute to the production of immunosuppressive factors IL-10 and TGF-β that can induce tolerogenic he-NK cells. Green arrows show stimulatory connection and red lines inhibition.

Human liver developed a high degree of immune tolerance as demonstrated by the clinical evidence indicating that liver allografts are less likely to be rejected than other transplanted organs (58). Several actors play different and fundamental roles in the maintenance of liver tolerogenic NK cells (Figure 2). KCs produce high doses of IL-10 which was observed to be critical in the control of mice intrahepatic NK cell-mediated alloreactivity (59). Indeed, an impaired ability of liver macrophages to produce this anti-inflammatory cytokine boost the IFN-γ-dependent priming of he-NK cells in response to double strand RNA exposure (60, 61). Moreover, the interplay between cNK cells co-cultured with human hepatic cells, and DCs induces the expansion of tolerogenic T cells (Tregs) via the engagement of CD94/NKG2A, which is a mechanism able to trigger the production of both transforming growth factor-β (TGF-β) and IL-10 (62, 63). Interestingly, *in vitro* stimulation of human cNK cells with apoptotic cells develops tolerance in these innate effector cells via the secretion of TGF-β that, in turn, suppresses their autocrine IFN-γ production (64).

Different studies demonstrated that he-NK cells are also important in regulating the unique capacity of liver to regenerate itself after tissue damage (65, 66). In this regard, in the *in vivo* model interaction of cNK cells with surrounding different liver-resident cells (i.e., KCs, fibroblast, and stem cells) induces the secretion of growth factors, hormones, cytokines, and chemokines able to induce the proliferation/regeneration of hepatic tissue (67). In particular, the activation of he-NK cells is associated with a *de novo* production of CXCL7, CXCL2, CCL5 and IL-8 that, in turn, can recruit and differentiate mesenchymal stem cells substantially contributing to the so-called “*restitutio ad integrum”* of this organ (65). This is a process that needs to be finely tuned and regulated since paradoxically over-stimulation of mouse he-NK cells can inhibit, rather than promoting, liver regeneration through the aberrant signaling pathway exerted by IFN-γ on those factors (i.e., STAT1, IRF-1, and p21cip1/waf1) regulating hepatocyte proliferation (68, 69). This is the case of *in vivo* activation with high doses of the immuno-stimulant Polyinosinic:polycytidylic acid (Poly I:C) (70).

## NK Cells in the Pathogenesis of Autoimmune Liver Diseases

Those mechanisms that make it possible for the liver to develop immunologic tolerance also expose this organ to the onset of immunological diseases. In this context, the presence of dysfunctional he-NK cells can actively contribute to the breach of immunological tolerance and in the appearance of autoimmune-liver diseases including autoimmune hepatitis (AIH), primary biliary cholangitis (PBC), and primary sclerosing cholangitis (PSC) (2, 71).

Although T cells have been reported to play a prominent role in the pathogenesis of AIH, several lines of evidence showed that also autoreactive he-NK cells are expanded in this autoimmune liver disorder (72). Indeed, the *in vivo* administration of Poly I:C in mice induces the onset of AIH in which activated intrahepatic NK cells actively contribute to liver damage (73). Additionally, the low frequency of the inhibitory KIR/KIR-ligand combinations KIR3DL1/HLA-Bw4 and KIR2DL3/HLA-C1 coupled to the high frequency of the HLA-C2 high affinity ligands for KIR2DS1 may contribute to unwanted NK cell autoreactivity in AIH (74). The expansion of aberrant NK cells able to kill autologous cholangiocytes represents also one of the pathogenic mechanisms present during the course of PBC (75, 76). Indeed, the frequency of he-CD56^dim^ NK cells in PBC is higher compared to that of healthy livers. However, it is still unclear whether the expansion of autoreactive he-NK cells targeting autologous biliary epithelial cells is directly associated with breach of liver immune tolerance or if this is a secondary event linked to the high degrees of immune activation and inflammation present in PBC (77). Another mechanism employed by cNK cells to lyse “self” cholangiocytes relies on the engagement of TRAIL pathway. As a matter of fact, the downstream death signal delivered by TRAIL receptor 5 is higher in PBC patients and induces cholestatic liver injury (78, 79). Another study also reported a protective role of intrahepatic NK cells in PBC patients, as the presence of low NK cell/cholangiocytes ratio is associated with higher IFN-γ production. This can induce or increases the expression of MHC-I and -II on cholangiocytes that are, in turn, spared from the lysis exerted by autoreactive NK cells. This latter protective mechanism is particularly relevant in the initial stages of PBC, since it can slow its progression to liver failure (80).

Among the three main liver autoimmune diseases, PSC represents the one whose pathogenesis is still largely unknown. However, the presence of certain HLA alleles or genetic variants of the NKG2D ligand MIC-A had been associated with higher risks of developing PBC. Indeed, both these molecular patterns regulate NK cell recognition of cholangiocytes (81). Similar to AIH and PBC, an increase of he-NK cell frequency was detected in PSC patients (82, 83). The most prominent current working hypothesis postulates that, similar to PBC, the engagement of TRAIL could induce the he-NK-mediated destruction of cholangiocytes in PSC patients (42, 79). Finally, another study reported that lr-NK cells from PSC patients are impaired in their cytotoxicity due to the high levels of local TNF-α production (84). Taken together, these contradictory data and speculations in regard to PSC pathogenesis reflect our general lack of knowledge in regard to the mechanistic roles and clinical impact of he-NK cells in liver autoimmune diseases.

## NK Cells in Liver Cancer

**Figure 3:**
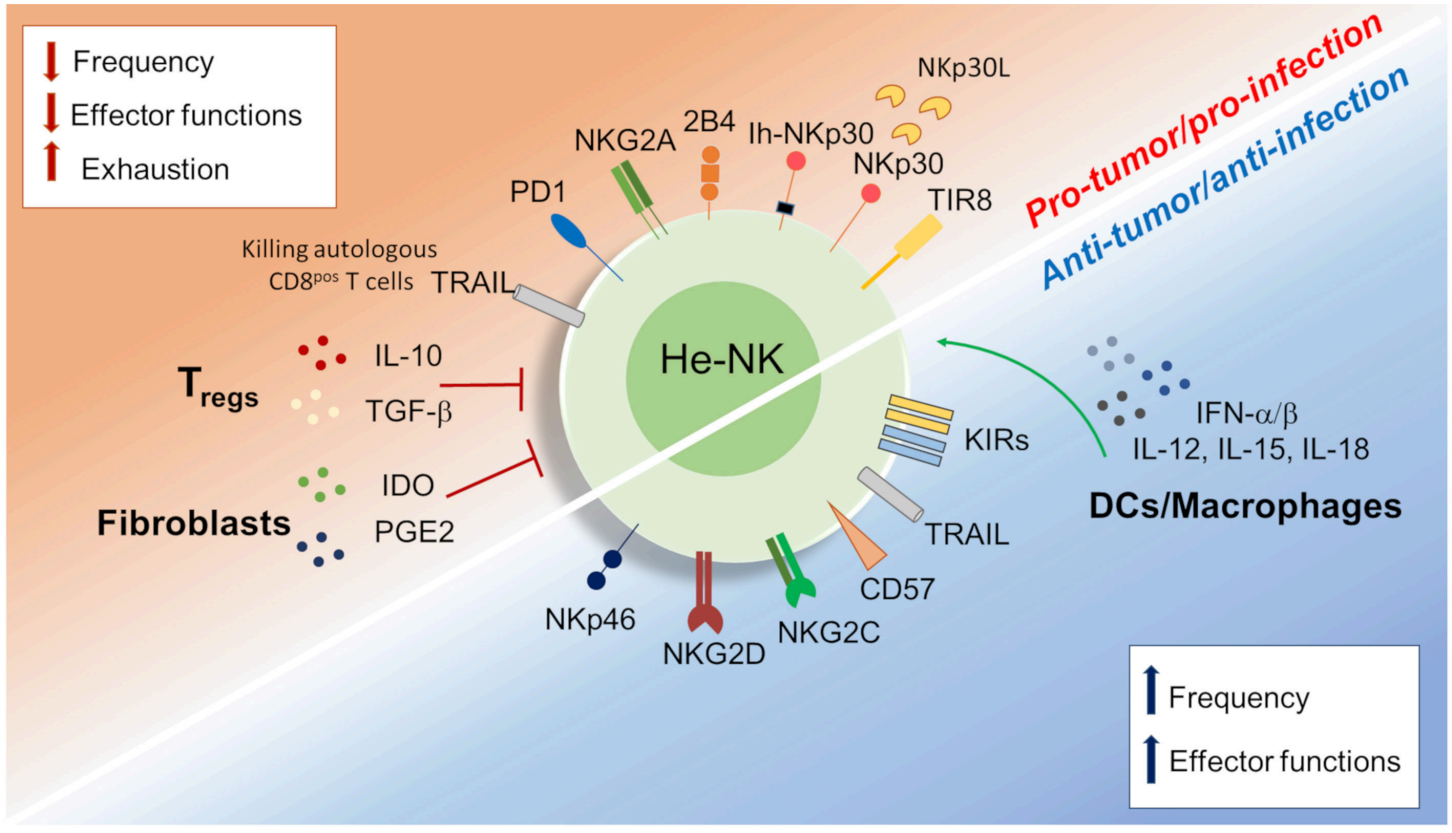
Dichotomy in the he-NK cell response in tumor and viral infection. Different soluble factors and cell surface receptors contribute to positive or either negative he-NK cell response in both tumor and/or viral infection. Increased (vertical blue arrows) frequency and effector-functions of he-NK cells were observed in early tumor and acute viral response. On the other hand, NK cell dysfunction (vertical red arrows) in late tumor or chronic infection results with lower NK cells frequency, effector function inhibition and cell exhaustion. Arched green arrows show stimulatory connection and red lines inhibition.

Hepatocellular carcinoma (HCC) is the most common leading cause of liver-cancer related death worldwide (85). Among the main predisposing risk factors of HCC, there are chronic viral infections by hepatitis B virus (HBV) and hepatitis C virus (HCV), alcohol related cirrhosis and non-alcoholic steato-hepatitis (86). The liver is also the first site of colorectal cancer (CRC) metastatic dissemination (87). NK cells had been first discovered due to their ability to kill tumor-transformed cells (i.e., immune surveillance) and are able to provide protection in hematological malignancies, solid primary cancers and metastatic lesions (88, 89). This important feature is also valid for HCC as human cNK cells were shown to be highly cytotoxic against HepG2 hepatocellular carcinoma cells (2, 42, 56). Moreover, it has been reported that higher numbers of total tumor infiltrating CD56^pos^ he-NK cells predict a better outcome for HCC in terms of patient overall survival (OS) (90–92). Other retrospective studies showed that high frequencies of the specific intra-tumor NK cell subsets slow the progression of liver cancer, as demonstrated for human NKp46^pos^ he-NK cells in CRC metastatic disease and for CD11b^neg^/CD27^neg^ he-NK cells in HCC (93, 94). In addition, the selective engagement of NKG2D in both mice and human enhances NK cell anti-tumor activity against HCC since the transcriptional modulation or the interferon-induced expression of this aNKR boosts he-NK cell cytotoxicity and blocks tumor growth (95, 96). This potent anti-tumor NK cell effector-function against HCC seems to be more effective in the early stages of the tumor and decreases as soon as the disease progresses (Figure 3). Indeed, lower frequencies of anergic/dysfunctional CD56^dim^ and CD56^bright^ NK cells, characterizes end-stages HCC patients both in peripheral blood and at a tumor site, a phenomenon that is also associated with a parallel expansion of CD4^pos^/CD25^pos^ Tregs and increased secretion of IL-10 (92, 97, 98). Several mechanisms have been proposed to explain, at least in part, the functional impairments of NK cells in advanced HCC. These include the increased expression on tumor infiltrating NK cell surface of inhibitory checkpoints [i.e., programmed cell death protein (PD-1) and NKG2A] as well as the higher surface levels of PD-1 ligands (PD-1Ls) and MHC-I on malignant cells. Both strategies simultaneously employed by HCC both on immune-effectors and targets have the same aim of evading human NK cell immune-surveillance, thus sparing tumor cells from NK cell killing (99–102). It has been also reported that in advanced HCC patients he-NK cells express a specific inhibitory NKp30 splice variant (Ih-NKp30), thus resulting in a deficiency of NKp30-mediated NK cell activation and function. Interestingly, the soluble form of NKp30 ligand (NKp30L) B7-H6 is increased in late stages of HCC (103). Another mechanism contributing to cNK cell impairment in HCC patients relies on their aberrant interactions with tumor infiltrating macrophages, inducing a rapid NK cell exhaustion both via the engagement of CD48/2B4 and NKp30 pathways (98, 104, 105). Additionally, several alterations in the cytokine milieu of neoplastic HCC tissue can influence cNK cell cytotoxicity and cytokine production. These include soluble immune-modulators such as TGF-β, prostaglandin E2 (PGE2) or indoleamine 2,3-dioxygenase (IDO) (105–107). More recently, it has been reported that IL-1R8 (TIR-8) can serve as another important checkpoint able to inhibit anti-tumor NK cell effector-functions in liver cancer murine models. Indeed, its blockade unleashes NK cell-mediated resistance to hepatic carcinogenesis and liver metastasis of CRC (108). Moreover, using a mouse model of cholangiocarcinoma (CCA), it has been demonstrated that adoptive NK cell transfer limits tumor growth and improves the prognosis of this aggressive liver cancer, although the related mechanisms associated to NK cell control of CCA have not yet been elucidated (109).

## Liver NK Cells and Viral Infection

HCV and HBV infections represent the main two infectious diseases inducing liver inflammation and failure (110). Controversial data are available regarding the immune status of he-NK cells in acute and chronic liver viral infections (Figure 3).

In acute HCV infection, he-NK cells show an increased expression of NKp46 and a high ability to degranulate and to produce IFN-γ following a strong activation by IFN-α/β and other cytokines (i.e., IL-12, IL-15, IL-18) (111, 112). Although intrahepatic CD56^bright^/NKp46^high^ he-NK cells contribute to a better control of HCV replication, their presence at high frequency is also associated with increased degrees of liver necrosis and fibrosis following acute infection (113). Interestingly, the livers of those patients experiencing self-limiting HCV-1 infection were not enriched of CD56^bright^/NKp46^high^ he-NK cells, but of NK cells are highly positive for CD57 and KIRs. These findings suggest that terminally-differentiated NK cells can better control HCV infection (114).

In the context of HBV infection, early cNK cell responses contribute to the initial control of infection and to the development of an efficient adaptive immune response through the secretion of IFN-γ, TNF-α, GM-CSF, and TGF-β able to inhibit viral replication or to induce the killing of infected cells (115–117). In this context, acute HBV infected patients showed an expansion of CD56^bright^ cNK subset, but reduced frequencies of CD56^dim^ NK cells. Notably, the inflamed lobular necrotic areas of HCV-infected livers from the same individuals were surrounded by NKp46^pos^ NK cells (118). *In vivo* experimental studies also confirmed the presence of a strong activation of he-NK cells in response to acute HBV infection, a mechanism that limits viral replication. However, in these animal models the HCV-mediated priming of NK cells was not able to induce an antigen-specific T cells response (119–121).

When both HBV and HCV enter into their chronic stages, the frequencies of cNK cells remarkably decrease together with their ability of producing pro-inflammatory cytokines, such as IFN-γ and TNF-α (122–125). Although, he-NK cells maintain their cytotoxic potential in chronic HBV via the up-regulation of TRAIL (126, 127), however, they pathologically contribute to eliminate autologous HBV-specific CD8^pos^ T cells expressing high levels of death receptor for TRAIL. Hence, this NK cell-mediated depletion of antigen-specific CD8^pos^ T cells impairs adaptive antiviral immunity in chronic HBV-infected patients and contributes to viral persistence (128–130). Moreover, persistent viral infections have a remarkable impact on the cNK cell receptor repertoire and profoundly affect their effector-functions. Indeed, chronic exposure to HBV induces TGF-β production that, in turn, reduces the expression of NKG2D and 2B4, and their respectively, intracellular adaptor proteins DAP10 and SAP, thus further hampering their ability to eliminate viral infected cells (131). However, whether this immunosuppressive mechanism plays a role in shaping he-NK cells need to be further consolidated.

## Hepatic NK Cells as Potential Therapeutic Targets

The possibility of tuning NK cell effector-functions represents an important therapeutic strategy for the treatment of several liver disorders, as demonstrated for infections and other malignancies (112, 113). Among the main methodological approaches developed in this context, there are protocols administering *in vivo* compounds targeting NK cell activation. Indeed, the use of several cytokines that can easily reach and activate liver endogenous NK cells has been extensively tested in several clinical and experimental trials. IL-12 and IL-18 have been shown to effectively inhibit liver carcinogenesis by boosting NK cell anti-tumor functions (132). Therapies with interferons showed anti-viral, anti-fibrotic and anti-tumor NK cell-mediated clinical outcomes (96, 133). Two cytokines widely adopted to enhance NK cell cytotoxicity are IL-2 and IL-15 (134, 135). In particular, IL-15 can rescue the anti-tumor activities of intrahepatic NK cells purified from HCC patients (136). Interestingly, the use of recombinant/modified IL-2 and IL-15 activates both NK and CD8^pos^ T cells without stimulating Tregs and these cytokines are currently being tested also against hematological cancers (137–139). Agonists for several aNKRs expressed on lr-NK cells, such as NKG2D and NCRs, also represent a potential clinical therapeutic strategy. Moreover, the expression of NKRs can be also modulated at the transcriptional level. In this regard, the miR-182 has been shown to increase NK cells cytotoxicity in HCC patients by regulating the expression of NKG2D and NKG2A (95).

In a new era of cancer immunotherapy, several inhibitory checkpoints have been targeted also on NK cells through the development of blocking mAbs unleashing their anti-tumor effector-functions (13). In particular, anti-KIR mAbs are currently being tested in different hematological cancers alone or in combination with other treatments (140). Another important NK cell inhibitory checkpoint is anti-NKG2A, whose masking mAb is currently being tested in several solid tumors and hematologic diseases (23, 141–144). More recently, it has been also reported that NK cells can express PD-1 thus paving the ground in the future to target NK cells also with mAbs blocking PD-1/PD-L1 interactions (145). Further clinical trials are required to investigate the efficacy of these compounds in liver cancers.

Adoptive NK cell transfer therapies have been first introduced to improve the clinical outcome of patients affected by hematologic malignancies and undergone allogeneic hematopoietic stem cell transplantation (allo-HSCT) (146, 147). The great clinical outcome of this strategy in allo-HSCT together with newly available technologies made it possible to develop new protocols of adoptive NK cell therapies to treat both hematologic malignancies and solid tumor (148–151). More recently, the possibility of engineering NK cells with different technological approaches such as the so-called bi- and tri-specific killer engagers (BiKEs and TriKEs) (152) or chimeric antigen receptors (CARs) (153) improved both tumor-specificity and the ability of NK cells to reach/infiltrate tumor tissues. Very little is known about the efficacies of adoptive NK cell transfer therapies in liver cancers, a gap that needs to be filled by new experimental and clinical trials.

## Concluding Remarks

Despite a great number of studies that have been focusing on elucidating the role of he-NK cells in liver physiology and physiopathology, several questions still remain unanswered. In particular, given the high heterogeneity of NK cells in liver, further studies are needed to investigate their specific role in both homeostatic and pathological conditions. Indeed, understanding this high degree of diversity will likely explain the several and often opposite functions of he-NK cells. These include the different capacities of he-NK cells either to reside in the liver or to recirculate through this organ without being retained and their abilities to be tolerogenic toward foreign antigens while attacking viruses and tumors. This knowledge is key to understand and target those mechanisms participating in the onset of hepatic disorders.

## Author Contributions

All authors listed have made a substantial, direct and intellectual contribution to the work, and approved it for publication.

### Conflict of Interest Statement

The authors declare that the research was conducted in the absence of any commercial or financial relationships that could be construed as a potential conflict of interest.
